# A framework for a teaching toolkit in entrepreneurship education

**DOI:** 10.1504/IJCEELL.2017.10003455

**Published:** 2017

**Authors:** Katharina Fellnhofer

**Affiliations:** LUT School of Business and Management, Lappeenranta University of Technology, P.O. Box 20, 53851 Lappeenranta, Finland

**Keywords:** education, entrepreneurial narratives, entrepreneurial storytelling, entrepreneurship, entrepreneurship education, multimedia teaching material, pedagogical toolkit

## Abstract

Despite mounting interest in entrepreneurship education (EE), innovative approaches such as multimedia, web-based toolkits including entrepreneurial storytelling have been largely ignored in the EE discipline. Therefore, this conceptual contribution introduces eight propositions as a fruitful basis for assessing a ‘learning-through-real-multimedia-entrepreneurial-narratives’ pedagogical approach. These recommendations prepare the grounds for a future, empirical investigation of this currently under-researched topic, which could be essential for multiple domains including academic, business and society.

## 1 Introduction

Because researchers are becoming increasingly interested in entrepreneurship education (EE) this emerging, multidisciplinary topic has been progressing with more high-quality reviews (Rasmussen, 2011; Lorz et al., 2013). While EE is recognised as a vital element for the whole economy among stakeholders (Schumpeter, 1934; Shane and Venkataraman, 2000), rising entrepreneurial awareness can act as a cornerstone in this development (e.g., Aviram, 2010; Fretschner and Weber, 2013; Levie et al., 2014). In particular, policy-makers consider EE a crucial basis for future economic development and growth [European Commission (EC), 2013a].

However, according to scholars, further research is required and essential for cultivating effective EE programs (e.g., Lorz et al., 2013; Bae et al., 2014). Several studies have acknowledged inconsistencies in findings regarding EE (Lorz et al., 2013; Rideout and Gray, 2013). Most research analysing EE focuses on the impact of variables such as skills and knowledge, attitudes and perceptions and intentions and performance (e.g., Lorz et al., 2013; Fellnhofer and Kraus, 2015; Fellnhofer, 2015, forthcoming). Despite a few exceptions to the rule (e.g., Oosterbeek et al., 2010), a preponderance of studies corroborate that EE has had a positive impact on individuals (e.g., Sternberg and Wennekers, 2005; Fayolle et al., 2006; Acs and Szerb, 2007; Souitaris et al., 2007). However, research done so far stresses disagreements on how to implement EE in practice (Kozlinska, 2011; Albornoz Pardo, 2013; Lourenço et al., 2013). Overall, there is no comprehensive, pedagogical toolkit to educate people in entrepreneurship (Neck and Greene, 2011). In general, EE challenges traditional, educational methods. In addition, for more than a whole decade there has been a serious call for novel and innovative entrepreneurial teaching techniques (Kuratko, 2003; EC, 2008, 2013c) as well as for more interactive and awareness-raising learning approaches (Kuratko, 2005; Miller et al., 2012; Neck and Greene, 2011; EC, 2013b). In this regard, Gendron (2004) as well as Lautenschläger and Haase (2011) suggest that there should be increased encouragement of entrepreneurs in class. This represents the point of departure for a multimedia approach to spread entrepreneurial narratives productively and resource-efficiently. Such a multimedia approach should utilise Sarasvathy’s (2001) theory of effectuation, which has directed lecturers to consider implementing the entrepreneur’s personal resources in the curriculum (Fletcher and Watson, 2007; Gibb, 2011). In line with this theory, EE has become continuously subject to a shift towards a more action-oriented, educational approach. This reallocation is based on a consensus in EE research that entrepreneurship is best imparted by using experimental, pedagogical tools (e.g., Fayolle et al., 2006; Noyes and Deligiannidis, 2013; Mayer et al., 2014; Assudani and Kilbourne, 2015). In light of this situation, this paper draws attention to the following research question: *Do multimedia entrepreneurial stories constitute an appropriate teaching tool to change perceptions towards entrepreneurship in a positive way?*

This qualitative, conceptual paper will first introduce the underlying framework. Within this approach, the propositions will be developed. Finally, a critical discussion will conclude this conceptual paper by taking due account of the limitations and practical and theoretical implications of the framework as well as making recommendations for future research.

## 2 The narrative framework

### 2.1 Entrepreneurial narratives for multimedia entrepreneurship education

From a pedagogical perspective, entrepreneurial role models need not be used in traditional, pedagogical methods such as teacher-learner interactions. Role models can be presented via multimedia through informal narratives with comparatively high value and impact with respect to low cost for introducing entrepreneurship (Kuratko, 2005). Stories – or narratives or storytelling – have been shown to have a significant impact on individual perceptions. Bruner (1986) claims that individuals classify familiarity and build reality in two basic ways: propositional judgment and narrative judgment. While the propositional method is based on a cause-to-effect analysis, narrative judgment includes information regarding persons, settings, intentions, and behaviour. Through these elements, narratives create a context to achieve high impact on individuals’ perceptions (Stewart, 1997). As indicated by Coles (1989), stories touch us and inspire us to take new paths. Moreover, stories tend to make us more open-minded. Therefore, entrepreneurial narratives are anticipated to be an effective educational approach for EE.

In response to a call to utilise the narrative approach (Johansson, 2004; Hamilton, 2006), research on entrepreneurial behaviour and motivational drivers have already experimented with this direction (Pless, 2007; Essers, 2009; Clarke and Holt, 2010; Flottemesch, 2013; Haley and Boje, 2014). Additionally, Kaminski (2003) recommends employing stories to teach leadership. Furthermore, Watson (2001) proposed using narratives including synthesised content from research and personal experiences to educate managers. The narrative approach has been also used to educate individuals in health and safety issues (Smith, 2005). While Friedman and Prusak (2008) illustrate the transdisciplinary, pedagogic value of narratives, which enhances and shapes our understanding of knowledge management, Gabriel and Connell (2010) discuss the pedagogic potential of narratives as a promising vehicle for management learning. Storytelling has also found its way into strategic management to support the introduction of change aspects and education of human resources (Swap et al., 2001; Sumner, 2005). Because the narrative approach has been successfully implemented in other pedagogical disciplines such as management learning and strategic management, the same is expected to hold true for EE.

The model of entrepreneurial potential elaborated by Krueger and Brazeal’s (1994) states that the emphasis on entrepreneurship in classrooms and proliferation of entrepreneurial knowledge, confidence building, and self-efficacy foster entrepreneurship as a socially accepted, highly regarded, personally rewarding and attractive career choice. For this reason, including stories of entrepreneurial role models in curricula show great potential as an effective tool to raise awareness of entrepreneurship as an attractive occupational path (Godsey and Sebora, 2009). Additionally, role models are living confirmation of attractive and achievable objectives, which support individuals to define their self-concept (Akerlof and Kranton, 2000) and develop self-efficacy to embark on an entrepreneurial career (Scherer et al., 1989; Krueger and Carsrud, 1993; Lockwood and Kunda, 1997; De Clercq and Arenius, 2006). Thus, entrepreneurial role models afford great potential to enhance aspirations for entrepreneurial activities (Arenius and De Clercq, 2005; Koellinger et al., 2007).

**Figure 1:**
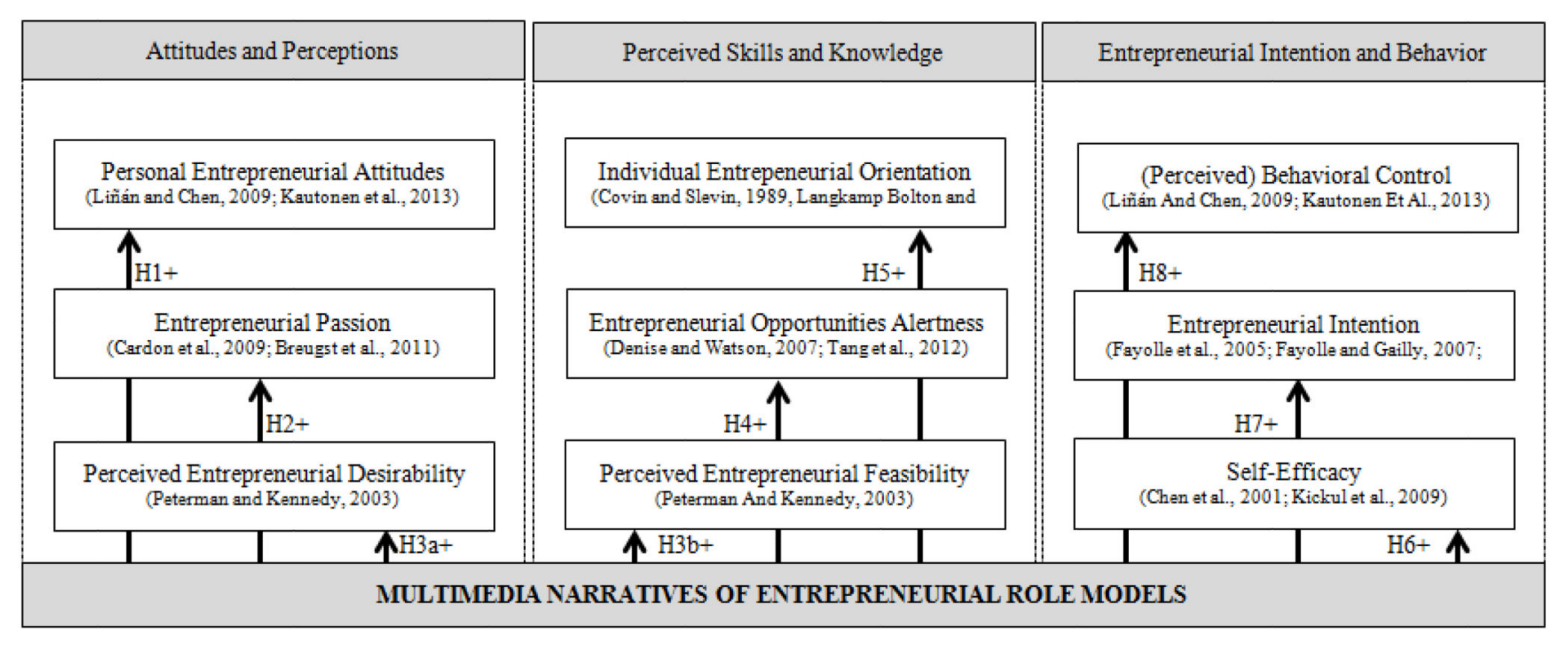
A conceptual model of expected impact variables

However, while in recent years entrepreneurship scholars have been encouraged to describe entrepreneurship from a life-story perspective, few scholars such as Robertson and Collins (2003), McAdams and Pals (2006), Engstrom (2012) or Flottemesch (2013) have used a self-narrative approach in EE. Narrative theory is often used for exploring and understanding new concepts. In particular, web-based storytelling has increasingly been used to accommodate several different learning styles (McAdams and Pals, 2006). By interviewing successful entrepreneurs and managers, Hood and Young (1993) suggest that practitioners’ recommendations, beliefs and experience should be included in EE curricula. In line with this suggestion and based on Boyles’ (2012) work, a conceptual model of expected impact variables as basis for this report’s propositions is presented in Figure 1. This study supports the grounds for highlighting that narratives enable potential entrepreneurs to:

1 Become motivated about setting up a company with respect to changing attitudes and perception in a positive way.
2 Become convinced in terms of perceived skills and knowledge to devote resources referring to entrepreneurial opportunities, in compliance with the results of Lurtz and Kreutzer (2014) with respect to feasibility.
3 Make key decisions linked to entrepreneurial intentions with respect to behaviour control.

Figure 1 provides the starting point for generating this report’s propositions. The variables for studying intended impacts of entrepreneurial narratives using multimedia approaches are outline below.

### 2.2 Eight impact variables as indicators for an adequate educational vehicle

#### 2.2.1 Impact on entrepreneurial attitudes

The key findings of Shinnar et al. (2009) and earlier results from Carsrud and Olm (1986) state that individuals’ views on entrepreneurship often differ. In particular, values and motivations play a crucial role in the intention-behaviour link (Fayolle et al., 2014). The high impact of beliefs about attitude, control, and subjective norms on behaviour mediated by intentions is captured by the Theory of Planned Behavior (TBP) (Ajzen, 1991). According to Ajzen (2011), intention is an individual’s eagerness to perform certain behaviour, and attitude refers to a person’s evaluation of this intended behaviour. Several researchers have evaluated the impact of EE on individuals’ entrepreneurial intentions through attitudes (e.g., Carayannis et al., 2003; Shinnar et al., 2009; Lourenço et al., 2013; Fayolle et al., 2014; Fellnhofer and Kraus, 2015). Several studies discovered that EE can have a positive impact on entrepreneurial attitudes by increasing the perceived attractiveness of entrepreneurship (e.g., Dickson, 2004; Krueger, 2007; Cheung, 2008; Petridou and Glaveli, 2008; Von Graevenitz et al., 2010). Further, researchers have shown that attitudes influence behaviours and behaviours influence attitudes (Brännback et al., 2007; Fretschner and Weber, 2013). This effect of attitude regarding entrepreneurship may vary regionally (Kautonen et al., 2013) or be influenced by culture (Liñán and Chen, 2009). In light of studies highlighting disparities in attitudes among persons who and were and were not participating in EE (Fayolle and Gailly, 2015), we assume that web-based entrepreneurial narratives support individuals in changing their entrepreneurial attitudes. Thus, we predict the following:

Proposition 1    Multimedia entrepreneurial narratives support individuals in changing their attitudes towards entrepreneurship.

#### 2.2.2 Impact on entrepreneurial passion

While feelings play a role in entrepreneurship (e.g., Baron, 2008; Foo et al., 2009; Cardon et al., 2012), in general entrepreneurship is linked with the exploration and exploitation of entrepreneurial opportunities (De Carolis and Saparito, 2006). Not only are strong, positive emotions fundamental in academic research on passion (e.g., Carver, 2003), but also in organisational psychology (e.g., Shipton et al., 2006; McMahon, 2009) and in the entrepreneurship literature (e.g., Baum and Locke, 2004; Chen et al., 2009; Cardon et al., 2013). Entrepreneurship is often faced with economic or social shifts and disruptive transformation (Metcalfe, 2004; Christensen et al., 2006), which create the need for continuous passion towards seeking entrepreneurial opportunities, developing ground-breaking products or services, and running initial prototypes while addressing the need for necessary resources (Cardon et al., 2009). Passion is not only desirable for finding a business (Chen et al., 2009), but also necessary for business development and enthusiasm to expand the enterprise (Gundry and Welsch, 2001). Based on these prior findings, we believe that narratives of passionate entrepreneurs can inspire their audience and therefore propose the following:

Proposition 2    Multimedia entrepreneurial narratives support individuals in enhancing their entrepreneurial passion.

#### 2.2.3 Impact on entrepreneurial perceived desirability and feasibility

According to the social psychology literature, intentions have been demonstrated to be effective predictors of planned behaviours (Krueger et al., 2000). The existing literature suggests two main antecedents of intentions, i.e. perceptions of desirability and feasibility (e.g., Shepherd and Krueger, 2002; Peterman and Kennedy, 2003; Kuehn, 2008; Armstrong, 2011; Wurthmann, 2014). In order to analyse these dependent drivers pre- and post-watching entrepreneurial narratives, this article suggests investigating these key variables – desirability and feasibility – as well. Many studies such as Ajzen (1991), Shapero (1975) and Shapero and Sokol (1982) discuss the origins of entrepreneurial intentions. In particular, Shapero and Sokol (1982) argue that entrepreneurial intentions originate from feasibility and desirability as well as perceptions and opportunity awareness, which are instrumental in any EE (De Clercq et al., 2013). Nonetheless, whether a generalisation to a narrative EE setting may occur is still an open question (Segal et al., 2005; Lanero et al., 2011). We propose that:

Proposition 3    Multimedia entrepreneurial narratives support individuals in changing their perceived entrepreneurial feasibility and desirability.

#### 2.2.4 Impact on opportunity exploration and exploitation

The exploration and exploitation of entrepreneurial opportunities act as the cornerstone of successful businesses (Sharma and Salvato, 2011; Gaimon and Bailey, 2013). Information and searching for information are key to recognising and exploiting business opportunities (Shane and Venkataraman, 2000; Fiet, 2007), and individuals can learn how to acknowledge patterns required to recognise these potential opportunities (Baron and Ensley, 2006). According to Patel and Fiet (2011), one can improve such recognition of opportunities. A study published by Heinonen and Poikkijoki (2006) related to opportunity discovery, evaluation, and exploitation provides information on applied teaching techniques in EE. This contribution summarises that the entrepreneurial-directed approach appears to be appropriate for broadening individuals’ perspectives and developing the skills for entrepreneurial behaviour. Based on Kirzner’s (1973, 1979, 1997) work regarding cognition theory and efforts by McMullen and Shepherd (2006), Tang et al. (2012) elaborated a reliable and valid model with three diverse elements of awareness including scanning and searching, relationship and connection, and evaluation and judgment. Given that this alertness scale can serve as a central indicator for effective EE, we propose the following:

Proposition 4    Multimedia entrepreneurial narratives support individuals in facilitating their entrepreneurial alertness related to opportunities.

#### 2.2.5 Impact on individual entrepreneurial orientation

The discussion around entrepreneurial orientation (EO) and its implications has received ample scholarly attention in past years (Rauch et al., 2009; Edmond and Wiklund, 2010; Covin and Lumpkin, 2011; Wales et al., 2013). While most academics view the EO construct as a firm-level phenomenon (Covin and Slevin, 1989b; Guth and Ginsberg, 1990; Zahra, 1993; Lumpkin and Dess, 1996; Antoncic and Hisrich, 2001, 2003), it can also be applied to the individual level, although few studies have implemented this approach (e.g., Poon et al., 2006; Joardar and Wu, 2011; Bolton, 2012; Langkamp Bolton and Lane, 2012; Goktan and Gupta, 2013). Based on the effort by Lumpkin and Dess (1996), Langkamp Bolton and Lane (2012) stress the reliability and validity of the used scale with 1,100 individuals. Weaver et al. (2002) applied also the scale of EO. Evidently, individuals are the reasons why enterprises operate entrepreneurially (Joardar and Wu, 2011; Covin and Miller, 2014). Since entrepreneurial role models highlight their EO in their narratives, we assume that entrepreneurs can have an impact on the EO of their audience. In light of the proposed implication, we infer the following:

Proposition 5    Multimedia entrepreneurial narratives support individuals in changing their EO.

#### 2.2.6 Impact on perceptions of entrepreneurial self-efficacy

Several researchers focus on evaluating the impact of EE on individuals’ intentions to develop a business on traditional antecedents of intentions such as self-efficacy (e.g., Cox et al., 2002; Maritz and Brown, 2013; Piperopoulos and Dimov, 2015). General self-efficacy refers to an individual’s estimation regarding his or her general ability to act successfully (Chen et al., 2001). At its heart, self-efficacy is not necessarily about entrepreneurial skills, but rather, one’s judgments of possessed skill capability. However, self-efficacy is still integral to entrepreneurship as many researchers have found that role models only inspire entrepreneurial intentions if they influence self-efficacy (Chen et al., 2001; Kickul et al., 2009). Because entrepreneurial narratives can increase the probability that individuals will consider an entrepreneurial career, such narratives most likely have a positive effect on individuals’ self-efficacy. In line with this argumentation, we assume the following:

Proposition 6    Multimedia entrepreneurial narratives support individuals in facilitating their entrepreneurial self-efficacy.

#### 2.2.7 Impact on entrepreneurial intentions

EE is expected to be associated with entrepreneurial self-efficacy, which may increase entrepreneurial intentions (e.g., Wilson et al., 2007; Liao and Gartner, 2008; Bae et al., 2014). There is a well-established research stream stressing that intentions prepare the grounds for founding a company (Liñán and Chen, 2009; Kautonen et al., 2013). Studies that examine EE’s impact on entrepreneurial intentions report positive outcomes (Lorz et al., 2013). As discussed, thus, we presume that the same holds true for a new innovative EE approach, and therefore, we predict:

Proposition 7    Multimedia entrepreneurial narratives support individuals in facilitating their entrepreneurial intentions.

#### 2.2.8 Impact on perceived behavioural control

In order to assess the effect of EE, most studies accept intention as the immediate determiner of behaviour (e.g., Krueger et al., 2000; Fayolle et al., 2006; Liñán and Chen, 2009). In particular, Kautonen et al. (2013) suggested modified questions and assertions (e.g., “I have applied much effort to activities aimed at starting a business in the last 12 months”.) to determine the link between intention and behaviour (Gollwitzer, 1999). In line with this argumentation, the following proposition requires examination when evaluating EE:

Proposition 8    Multimedia entrepreneurial narratives support individuals in facilitating their perceived behavioural control.

## 3 Discussion and conclusions

With respect to recommendations by Lorz et al. (2013), essential elements have been discussed in this conceptual paper as a basis for the future measurement of real entrepreneur narratives as an awareness-raising toolkit using multimedia. Based on the methods of other impact studies (e.g., Kolvereid and Moen, 1997; Wilson et al., 2007; Liao and Gartner, 2008; Olomi and Sinyamule, 2009; Kružić and Bulog, 2010), a structured sampling procedure with an adequate sample size for an ex ante/ex post design represents a further essential requirement for evaluating entrepreneurial narratives as an adequate teaching tool (Lorz et al. 2013). In addition, because most EE studies are carried out at higher educational levels (e.g., Vanevenhoven and Liguori, 2013; Volery et al., 2013; Zhang et al., 2014), a focus on currently under-researched target groups such as students of vocational schools (Lorz et al., 2013) will enrich the academic discussion. Only few studies have explored the impact of EE on these groups (Peterman and Kennedy, 2003; Athayde, 2009; Oosterbeek et al., 2010).

As the worldwide dissemination of EE continues (Solomon and Fernald, 1991; Edelman et al., 2008; Joshi and Ganapathi, 2008; Kailer, 2009; Ion and Viorica, 2011; Boyles, 2012) along with growing academic interest in its role and impact (DeJaeghere and Baxter, 2014; Saeed et al., 2014; Sipon and Lope Pihie, 2014; Fayolle and Gailly, 2015), there appears to be little agreement regarding what the content of EE should be as well as how to implement teaching tools in these programs (Honig et al., 2005; Neergaard and Ulhøi, 2007; Edelman et al., 2008). In particular, researchers have paid little attention to the impact of entrepreneurial narratives on individuals (Davidsson, 2006; Pittaway and Cope, 2007; Xavier et al., 2008; de Vries, 2014). In response to this under-researched, phenomenal development and promising topic, this article draws attention to essential impact indicators for a narrative teaching approach within the field of EE privilege. Eight propositions are suggested for a framework to demonstrate that an entrepreneurial narrative approach in EE will produce a positive impact on entrepreneurial attitudes (Proposition 1), entrepreneurial passion (Propositions 2), perceived desirability and feasibility (Propositions 3), alertness to entrepreneurial opportunities (Propositions 4), one’s EO (Propositions 5), perceptions of entrepreneurial self-efficacy (Propositions 6), entrepreneurial intention (Propositions 7) and, finally, perceived behavioural control (Propositions 8). Overall, this report stresses that the multimedia entrepreneurial narrative approach affords great potential impact on multiple stakeholders. We strongly suggest that entrepreneurial storytelling could be a useful tool to shape and foster entrepreneurship as an attractive career path. However, this perspective needs to be cast in a formal model with empirical data to ascertain if real entrepreneurial stories support individuals changing their perspectives towards entrepreneurship in a positive way. The proposed framework provides a basis for future research in this discipline.
